# The development and use of an ELISA-based method to follow the distribution of cellulase monocomponents during the hydrolysis of pretreated corn stover

**DOI:** 10.1186/1754-6834-6-80

**Published:** 2013-05-20

**Authors:** Amadeus Y Pribowo, Jinguang Hu, Valdeir Arantes, Jack N Saddler

**Affiliations:** 1 Forest Products Biotechnology/Bioenergy Group, University of British Columbia, 2424 Main Mall, Vancouver, British Columbia, V6T1Z4, Canada

**Keywords:** Cellulose, Cellulase, Enzyme, Adsorption, ELISA

## Abstract

**Background:**

It is widely recognised that fast, effective hydrolysis of pretreated lignocellulosic substrates requires the synergistic action of multiple types of hydrolytic and some non-hydrolytic proteins. However, due to the complexity of the enzyme mixture, the enzymes interaction with and interference from the substrate and a lack of specific methods to follow the distribution of individual enzymes during hydrolysis, most of enzyme-substrate interaction studies have used purified enzymes and pure cellulose model substrates. As the enzymes present in a typical “cellulase mixture” need to work cooperatively to achieve effective hydrolysis, the action of one enzyme is likely to influence the behaviour of others. The action of the enzymes will be further influenced by the nature of the lignocellulosic substrate. Therefore, it would be beneficial if a method could be developed that allowed us to follow some of the individual enzymes present in a cellulase mixture during hydrolysis of more commercially realistic biomass substrates.

**Results:**

A high throughput immunoassay that could quantitatively and specifically follow individual cellulase enzymes during hydrolysis was developed. Using monoclonal and polyclonal antibodies (MAb and PAb, respectively), a double-antibody sandwich enzyme-linked immunosorbent assay (ELISA) was developed to specifically quantify cellulase enzymes from *Trichoderma reesei*: cellobiohydrolase I (Cel7A), cellobiohydrolase II (Cel6A), and endoglucanase I (Cel7B). The interference from substrate materials present in lignocellulosic supernatants could be minimized by dilution.

**Conclusion:**

A double-antibody sandwich ELISA was able to detect and quantify individual enzymes when present in cellulase mixtures. The assay was sensitive over a range of relatively low enzyme concentration (0 – 1 μg/ml), provided the enzymes were first pH adjusted and heat treated to increase their antigenicity. The immunoassay was employed to quantitatively monitor the adsorption of cellulase monocomponents, Cel7A, Cel6A, and Cel7B, that were present in both Celluclast and Accellerase 1000, during the hydrolysis of steam-pretreated corn stover (SPCS). All three enzymes exhibited different individual adsorption profiles. The specific and quantitative adsorption profiles observed with the ELISA method were in agreement with earlier work where more labour intensive enzyme assay techniques were used.

## Introduction

One of the key steps in a biomass-to-ethanol process is the enzymatic hydrolysis of the cellulosic component to fermentable sugars. Typically, a mixture of complementary cellulase and other, so-called, accessory enzymes (such as hemicellulases, GH61, etc.) are required to effectively break down the structural cellulose and hemicellulose polysaccharides to their component sugars [1,2]. However, various technoeconomic analyses have indicated that the cost of enzymatic hydrolysis is still unacceptably high, primarily because of the high enzyme loadings required to achieve effective hydrolysis [3]. As a result, a considerable amount of research has focussed on ways to try to improve the efficiency of hydrolysis while using low protein/enzyme loadings. Various strategies have been assessed, such as increasing substrate digestibility through biomass pretreatments [4,5], improving the efficiency of enzyme cocktails [6,7], and reusing the enzymes for multiple rounds of hydrolysis [8,9]. The last two strategies, in particular, have benefitted from better characterization of the specific roles and actions of individual enzymes and their synergistic interaction during cellulose hydrolysis.

However, getting a better understanding of the individual enzyme’s interaction with the substrate during hydrolysis of lignocellulosic substrates has been challenging, primarily because of the lack of specific techniques that can overcome both the complexity of the enzyme mixture and the interference caused by the heterogeneous lignocellulosic substrates. Many of the biochemical techniques that might be used lack the resolution to specifically probe individual enzymes and proteins. For example, the enzyme Cel7A from *T. reesei* has a very similar molecular weight to that of Cel6A and Cel7B and, consequently, these three enzymes typically show up as a single band after gel electrophoresis [1]. Another commonly used technique is to characterize and evaluate distribution of enzymes based on their activities on model substrates such as carboxymethyl cellulose (CMC), filter paper, or a number of chromophoric substrates such as p-Nitrophenyl-based substrates [10]. Unfortunately, many of these model substrates are not specific enough to distinguish individual enzymes. Protein chromatography techniques have also been utilized to fractionate the enzyme mixture down to its individual components [11,12]. However, this approach is laborious and, depending on the purification protocols used, the enzyme mixture may not always completely separate into its individual components [13]. In addition, interference caused by substrate materials such as lignin auto-fluorescence limits the use of traditional protein chromatography techniques and protein labelling techniques using fluorescent dyes [14].

Primarily due to the limitations of the assay methods that have been employed, most of the previous enzyme-cellulosic substrate interaction studies have used purified enzymes or reconstituted mixtures of purified enzymes [15,16] and/ or model substrates such as pure cellulose or substrates with a very low lignin content [17,18] to simplify the subsequent enzyme assays and analyses. While these studies have advanced our understanding of enzymes-substrate interaction, they have not looked at the interactions occurring during the hydrolysis of an industrially relevant lignocellulosic substrate using a complete enzyme mixture.

In recent work, the distribution of individual enzymes present in a commercial cellulase mixture (Accellerase 1000) was assessed during the hydrolysis of steam pretreated corn stover (SPCS) [1]. A combination of methods such as, gel electrophoresis, zymograms, activity assays using chromophoric substrates, and mass spectrometry were used to define the general distribution patterns of some of the enzymes during SPCS hydrolysis [1]. However, although we were able to semi-quantitatively assess enzyme distribution using these techniques, we were not able to quantitatively follow the adsorption profiles of individual enzymes.

It is well known that antibodies can bind to specific antigens and this ability has been used as the basis for many assays [19-21]. This specific recognition and binding has been utilized in various techniques including the enzyme-linked immunosorbent assay (ELISA). The ELISA method uses antibodies linked to a reporter enzyme to specifically recognize and bind a target compound in a mixture of compounds. This specific compound or protein can then be quantified by adding a substrate for the reporter enzyme and measuring the concentration of the product [22]. The ELISA method, using monoclonal and/or polyclonal antibodies (MAbs and PAbs, respectively) raised against various cellulase enzymes, has been successfully used to quantify target enzymes both in culture filtrates and commercial enzyme preparations [19].

A double-antibody sandwich ELISA, which is an ELISA-based technique using a pair of antibodies to sandwich the target compound and specifically quantify it among other compounds in the mixture, has been successfully used to quantify the amount of Cel7A in a crude culture broth with minimal interference from other enzymes or other materials present in the broth [23]. Improved specificity of the assay was achieved when MAb was used as the coating antibody and PAb as the second, detecting antibody [23]. In related work, Buhler et al. (1991) optimized a double-antibody sandwich ELISA for Cel7B in a culture broth using MAb as the coating antibody and PAb as the detecting antibody. They were able to show that the assay was both sensitive and specific for Cel7B [24]. However, the feasibility of using ELISA to quantify specific proteins present in the supernatant after hydrolysis of a lignocellulosic substrate has not yet been demonstrated.

In the work described here, a double-antibody sandwich ELISA was developed and used to quantify some of the specific cellulase enzymes present in the supernatant during the hydrolysis of SPCS. A double-antibody sandwich  ELISA was used to specifically quantify the amount of cellulase monocomponents Cel7A, Cel6A, and Cel7B present in a commercial enzyme mixture. The sensitivity was improved by subjecting the enzyme samples to a pH adjustment treatment and/or a heat treatment. While lignocellulosic substrate derived materials did interfere with the assay, this interference could be minimized by simple dilution.

## Results and discussions

### Determination of the specificity of the different monoclonal antibodies (MAbs) and polyclonal antibodies (PAbs)

**Figure 1:**
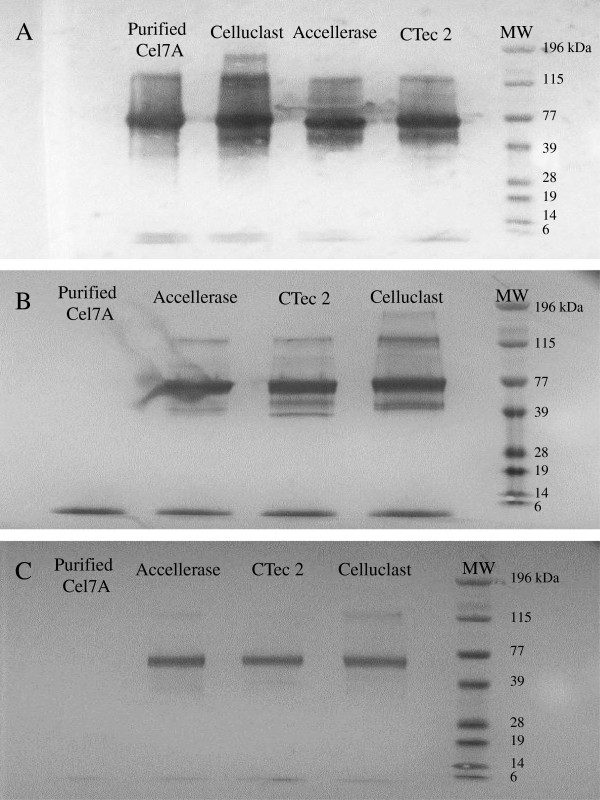
Reactivity and specificity of MAbs against Cel7A (A), Cel6A (B), and Cel7B (C) as determined using Western Blots.

We initially wanted to ensure that the MAb and PAb that we had been provided were specific for their target cellulase monocomponents. The specificity of Cel7A, Cel6A, and Cel7B MAbs were initially assessed using Western Blots against Cel7A that had been purified from a commercial Celluclast mixture as well as against the Cel7A component that was known to be present in the 3 commercial enzyme mixtures. The Cel7A MAb Western Blot showed a single band corresponding to the purified Cel7A and a major band at molecular weight (MW) ~ 70 kDa, which is the molecular weight of the Cel7A, present in the 3 commercial enzyme mixtures (Figure 1A). Although the Cel6A MAb also showed a band of protein at MW ~70 kDa when assayed against the 3 commercial enzyme mixtures (Figure 1B), this MAb did not react with the purified Cel7A. In addition to the major bands at MW ~70 kDa, Cel7A and Cel6A MAb Western Blots both showed multiple bands with commercial enzyme mixtures. Although we could not be certain if these bands corresponded to multiple isoforms of the target enzyme or actual unspecific bindings to other proteins without further experiments, we did not expect these apparent multiple bindings to significantly influence the specificity of the ELISA for 2 reasons. Firstly, the intensity of these other bands was significantly less compared to the band intensity of the expected target enzyme. Therefore, given the low protein concentration required for ELISA (< 5 μg/ml), this apparent unspecific binding (if any) would not likely to have any significant influence to the specificity of the assay. Secondly, the differing banding patterns between Cel7A and Cel6A Western Blots seemed to suggest a specific rather than an unspecific binding. The Western Blot that used the Cel7B MAb did not recognize the purified Cel7A but recognized a band of protein at MW ~60 kDa in all of the 3 commercial enzyme mixtures (Figure 1C). Therefore, it appears that all 3 MAbs were reactive and specific for their target enzymes.

**Figure 2:**
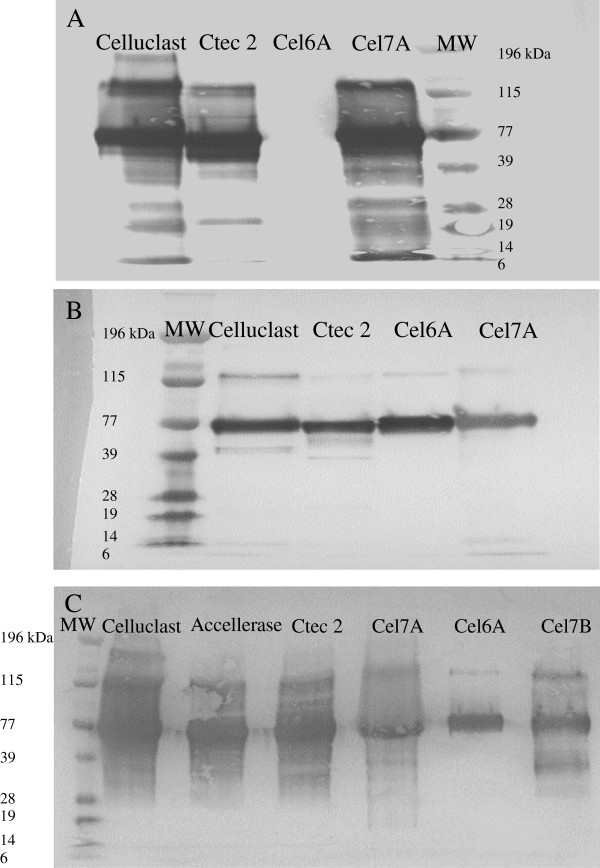
Reactivity and specificity of PAb against Cel7a (A), Cel6A (B), and Cel7B (C) as determined using Western Blots.

The specificity and reactivity of Cel7A and Cel6A PAbs were also determined by Western Blots by using purified Cel7A and Cel6A from Celluclast as well as 2 commercial enzyme mixtures. The PAb against Cel7A was specific for its target enzyme since it reacted only with purified Cel7A and not with the purified Cel6A (Figure 2A). However, the PAb against Cel6A recognized both the purified Cel7A and Cel6A (Figure 2B). Possible contamination by Cel6A in the purified Cel7A fraction did not appear to be an issue as the Cel6A MAb did not react with the purified Cel7A preparation (Figure 1B). The reactivity and specificity of the Cel7B PAb was next determined using Western Blots against purified Cel7A, Cel6A, and Cel7B as well as against 3 commercial cellulase mixtures. It was apparent that the Cel7B PAb recognized the purified Cel7B but also cross-reacted with the purified Cel7A and Cel6A (Figure 2C). However, this cross-reactivity with the Cel6A and Cel7B PAbs was not expected to influence the specificity of the double-antibody sandwich ELISA since both the Cel6A and Cel7B MAbs were shown to be specific to their respective target enzymes (Figure 1B and C).

### Optimization of the assay protocols to improve the sensitivity of the double-antibody sandwich ELISA

Previous work had shown that a double-antibody sandwich ELISA, using a combination of a MAb and a PAb as the capture and detecting antibodies respectively, resulted in improved specificity compared to normal ELISA or to a sandwich ELISA using PAb as the capture and MAb as the detecting antibody [19,23]. Thus, we next used an MAb as the capture antibody and a PAb as the detecting antibody to assay different concentrations of each of the 3 antibodies MAb, PAb, and goat-anti rabbit IgG conjugated to alkaline phosphatase (GAR-AP). In this way, we hoped to assess the sensitivity of the assay in detecting purified Cel7A at concentrations ranging from 0–2.5 μg/ml.

**Figure 3:**
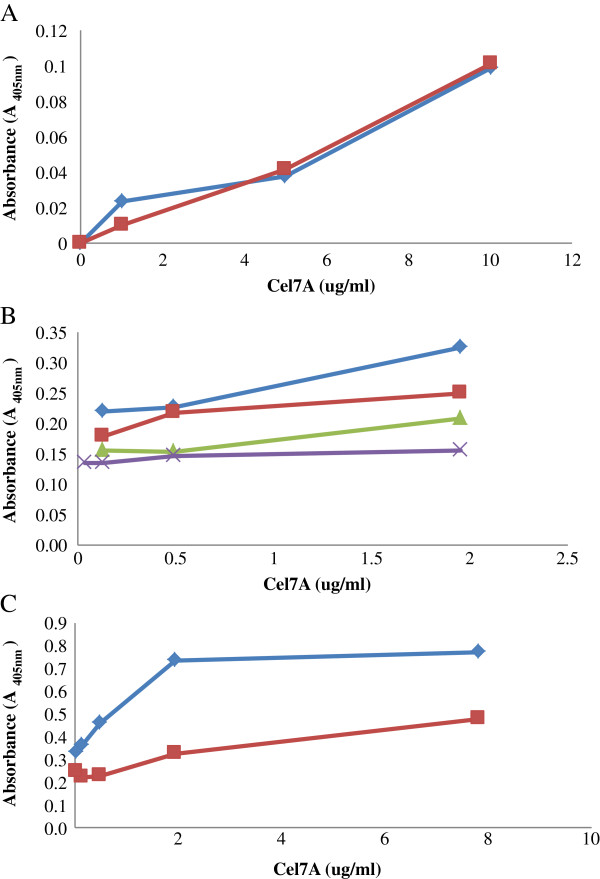
**Optimization of the concentrations of MAb (A), PAb (B), and the third antibody, GAR-AP (C) over a range of concentration of purified Cel7A.** (**A**). Two different concentrations of Cel7A MAb 10 μg/ml (◊) and 50 μg/ml (□) were added to the well. PAb and GAR-AP concentrations were kept constant at 1/400 and 1/1750 dilutions, respectively (**B**). Using 10 μg/ml Cel7A MAb, the Cel7A PAb was diluted to different degrees: 14(◊), 7(□), 3.5(∆), and 1.75(X) μg/ml. The GAR-AP was diluted at 0.3 μg/ml. (**C**). The concentration of GAR-AP was varied by diluting it to 1 (◊) or 0.3 (□)μg/ml in PBS. Cel7A MAb concentration was kept at 10 μg/ml, and Cel7A PAb was diluted to 14 μg/ml in PBS.

Although two concentrations of Cel7A MAb (10 and 50 μg/ml diluted in 1x Phosphate-Buffered Saline or PBS) were initially assessed, as both concentrations gave similar absorbance values (Figure 3A), a MAb concentration of 10 μg/ml was used in subsequent work. Previous work had also determined that a concentration of 10 μg/ml was sufficient to coat the bottom surface of a well in a typical 96-well ELISA plate [25]. The concentrations of the PAb (detecting antibody) and GAR-AP, the tertiary antibody, were similarly optimized over the same range of Cel7A concentrations. A concentration of 0.14 μg/ml of PAb Cel7A and 1/500 dilution of GAR-AP (corresponding to 1 μg/ml of GAR-AP) were found to improve the sensitivity of the assay for all the three enzymes (Figure 3B and C). These concentrations of antibodies were then also used for the Cel6A and Cel7B based ELISA’s.

Despite the increased sensitivity gained by optimizing the concentrations of all 3 antibodies, the improved signal was still quite low when compared to previously reported values [23]. Therefore, to try to further increase the sensitivity of the assay, the enzyme samples were subjected to pH adjustment and heat treatments prior to addition to the well. Although previous work had shown that the antigen-antibody interactions are typically optimum at pH > 7 [24], fungal derived enzymes are typically buffered and used at around pH < 5. We therefore brought the enzyme samples up to pH 7.5 using PBS buffer prior to their addition to the wells.

**Figure 4:**
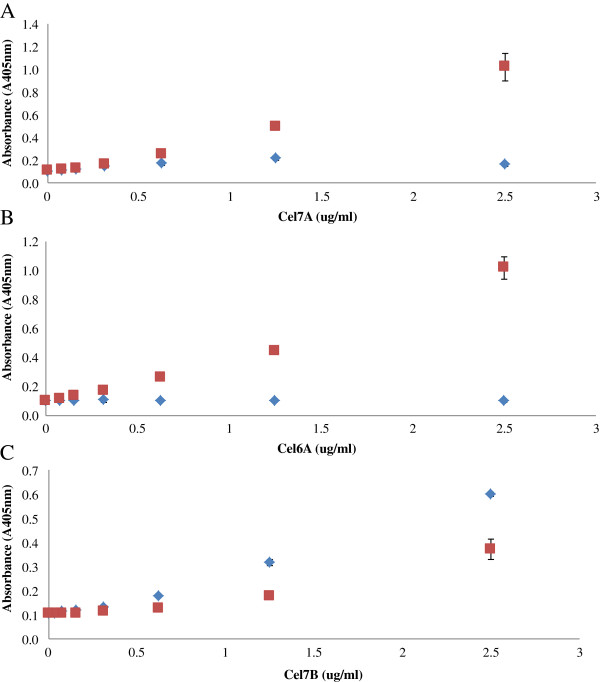
**The effect of a heat treatment on the sensitivity of ELISA for pure Cel7A (A) and Cel6A (B) and Cel7B (C).** Heated enzyme samples were boiled in Na-acetate buffer pH 5.0 at 100°C for 10 minutes and then serially diluted in PBS (□). Non-heated samples were directly diluted in PBS (◊).

Previously, Riske et al. (1990) had reported that a heat-sensitive fungal product caused a signal reduction with Cel7A ELISA and that this interference disappeared after the cellulase preparation was boiled, resulting in increased ELISA sensitivity [23]. Therefore, to see if we could also obtain the same beneficial effect, the enzyme monocomponents were also heated at 100°C for 10 minutes to determine if a heat treatment might also improve sensitivity. When the Cel7A and Cel6A ELISA’s were subjected to a heat treatment at 100°C for 10 minutes in a pH 5.0 buffer, followed by dilution in PBS buffer at pH 7.5, the sensitivity of ELISA increased by about 6× and 10× respectively for Cel7A and Cel6A, at an enzyme concentration of 2.5 μg/ml when compared to the untreated samples (Figure 4A and B). However, heat treatment decreased the sensitivity for the Cel7B based ELISA (Figure 4C). Therefore, the enzyme samples for the Cel7B ELISA were not heated but directly diluted in PBS buffer and then added to the wells.

As mentioned earlier, the improved signal achieved by heating the enzymes used for the Cel7A and Cel6A based ELISA’s was likely caused by the removal of interfering heat-sensitive materials present in the samples [23] or by protein denaturation which may lead to the opening up of the protein structure, exposing the antigen to the antibody. The ineffectiveness of heating the Cel7B may indicate that the interfering materials may not interfere with the Cel7B based ELISA system. This differential response to the heat treatment highlights the need to optimize the double-antibody sandwich ELISA for each specific enzyme-antibody system.

### How specific is the ELISA to the enzyme of interest?

**Figure 5:**
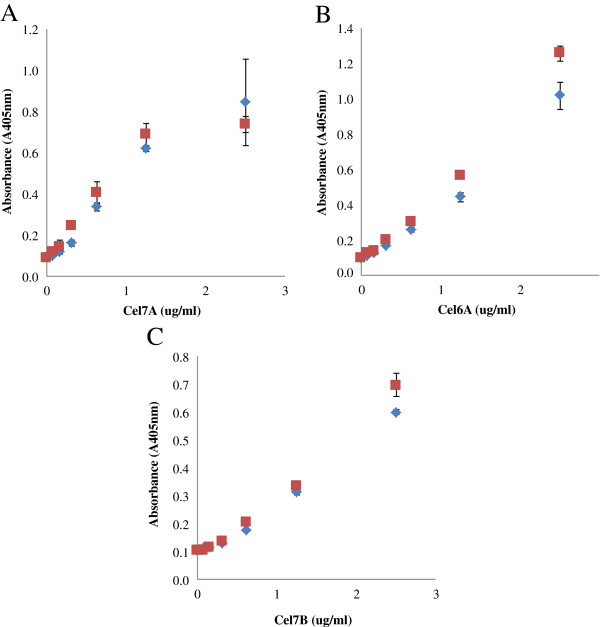
The specificity of ELISA for Cel7A (A), Cel6A (B), and Cel6A (C) as measured using pure enzymes (◊) and reconstituted mixtures of the 4 purified enzymes Cel7A, Cel6A, Cel7B, and Cel5A (□).

The specificity of each ELISA was next determined by comparing the absorbance values of each enzyme when it was added as a single component and when it was added as a mixture of 4 purified enzymes (Cel7A, Cel6A, Cel7B, and Cel5A). For all of the enzyme based ELISA’s (Cel7A, Cel6A, and Cel7B ELISA), the standard curves obtained with the pure enzymes was similar to those obtained with the reconstituted mixture especially when the target enzyme concentration was less than 1 μg/ml (Figure 5A, B, and C). It was apparent that the ELISA double-antibody sandwich assay was able to specifically quantify a target enzyme when it was present in a mixture with 3 other cellulase monocomponents.

**Figure 6:**
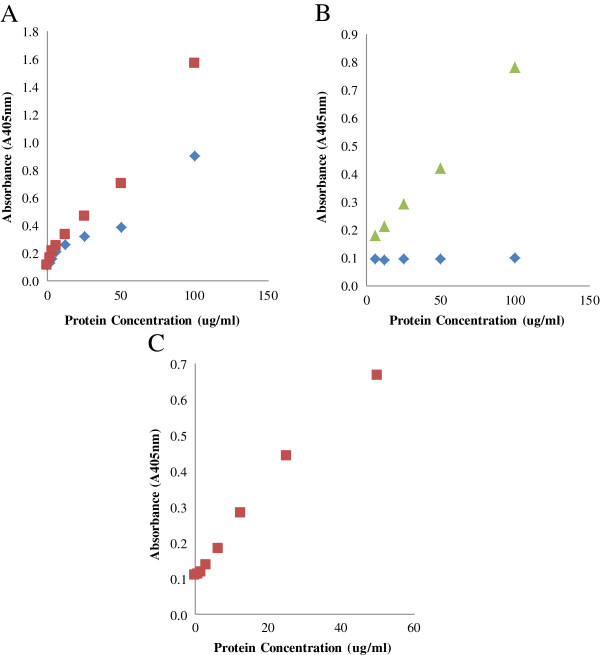
The construction of a standard curve for Cel7A ELISA (A) using whole commercial enzyme mixtures Accellerase 1000 (◊), CTec 2 (□), Cel6A ELISA (B) using Accellerase 1000 (◊) and Celluclast 1.5L (∆), and Cel7B ELISA (C) using CTec 2 (□).

We next wanted to determine if a whole commercial enzyme mixture could be used to make a standard curve, thus obviating the need for purified enzymes. A commercial enzyme mixture was diluted to 200 μg protein/ml in Na-acetate buffer (0.05 M, pH 5.0). When using the Cel7A and Cel6A based ELISA’s, the commercial enzyme mixtures were heated, serially diluted 2-fold in PBS and then added to the wells. By sufficiently diluting the enzyme mixtures, a relatively linear standard curve could be obtained with whole enzyme mixtures when using the Cel7A and Cel7B based ELISA’s (Figure 6A and C). A linear standard curve was also obtained with the Cel6A ELISA. However, this linear standard curve was only obtained with Celluclast and not with Accellerase or CTec 2 (Figure 6B).

The linear standard curve obtained for all of the target enzymes highlighted the ability of the double-antibody sandwich ELISA to detect the target enzyme even when present in complex enzyme mixtures. The high specificity of the MAbs could also be the reason why Cel6A ELISA only worked with Celluclast and not with other commercial enzyme mixtures as the Cel6A MAb was developed by colleagues at the National Renewable Energy Laboratory (NREL) to detect Cel6A in Celluclast whereas the PAb was developed commercially by Alpha Diagnostics using a synthesized peptide. Although both the MAb and PAb’s against Cel6A recognized the Cel6A present in Celluclast, Accellerase and CTec 2 (Figure 1B and 2B), the lower ELISA signal observed in the latter two commercial enzyme mixtures might be a result of a slight change in antigen recognition by the MAb. When the concentration of the enzymes and antibodies are high, as in the case of the Western Blot studies (30 μg of enzyme samples and 250 μg MAb or PAb), there is likely enough interaction between the enzymes and antibodies, resulting in a significant band on the membrane. However, when the enzyme concentration is low (< 0.1 μg), as in the case with the ELISA, the lower binding affinity between the antibodies and Cel6A in Accellerase and CTec 2 would result in a lower ELISA signal. It was apparent that a double-antibody sandwich ELISA was specific for target enzymes providing appropriate MAbs and PAbs were available. Given the recent rapid development of enzyme cocktails to which new-and-improved enzymes have been introduced, (i.e. CTec3) the highly specific nature of the antibody-antigen interaction shown in this assay will likely require the development of specific MAbs and PAbs that will recognize individual enzymes present in these new and improved enzyme mixtures.

### Determining the possible interference of substrate derived materials on the ELISA

Although various ELISA based methods have been used to quantify cellulase enzymes, these assays have only been applied to commercial enzyme mixtures or to culture filtrates [19,23,24,26]. The use of an ELISA to try to follow the distribution of cellulase enzymes during enzymatic hydrolysis of a realistic, lignocellulosic substrate has not, so far, been described in the literature As a result, there is limited information on the possible influence of interfering materials that will likely be present when attempts are made to use an ELISA in this situation.

Previous work on the use of ELISA’s to detect residual agrochemicals in soil samples had shown that humic substances in soil may result in an overestimation of chemical concentrations [20,27,28], and that sample dilution could be used to minimize interference [20]. As a similar type of interference might occur with biomass-derived materials such as soluble lignin fragments, supernatants derived from steam pretreated corn stover (SPCS), steam pretreated poplar (SPP), steam pretreated douglas fir (SPDF), and Avicel were assessed for their possible influence on the double-antibody ELISA. The supernatants were diluted in PBS to varying degrees to determine if a simple dilution could minimize the interference caused by these materials.

**Figure 7:**
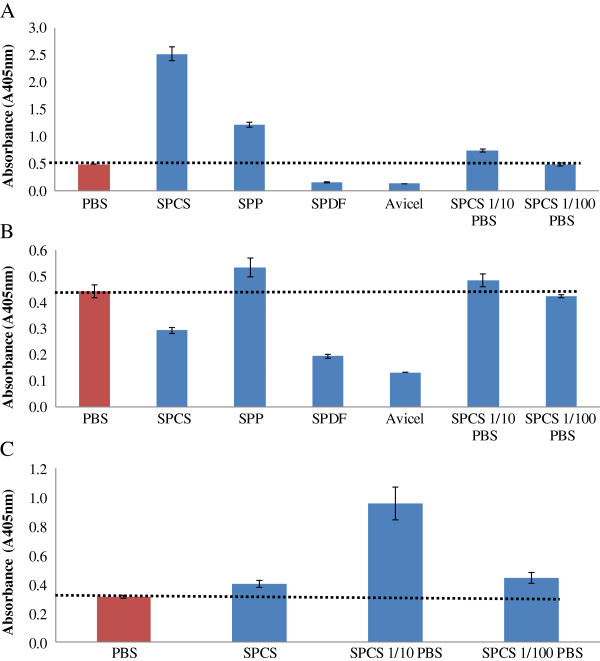
**Effect of substrate supernatants on Cel7A ELISA (A), Cel6A ELISA (B), and Cel7B ELISA (C).** Amount of purified enzymes added: 1.25 μg/ml.

It was apparent that the undiluted biomass derived supernatants resulted in considerable interference with all of the Cel7A, Cel6A, and Cel7B based ELISAs (Figure 7). The Cel7A ELISA either over or under estimated the amount of enzyme (Figure 7A) with the supernatants derived from the SPCS (5× higher) and SPP substrates resulting in an overestimation and the SPDF and Avicel supernatants in an underestimation (Figure 7A). In contrast, only the SPP supernatants caused a signal overestimation with Cel6A ELISA while the SPCS, SPDF, and Avicel supernatants gave a signal that was lower than the PBS control (Figure 7B). Interference with Cel7B based ELISA was only assessed with the SPCS supernatant which caused a slight overestimation (Figure 7C). To assess if a simple dilution could minimize interference, each supernatant was diluted 10× or 100× in PBS. It was apparent that the interference caused by the addition of the undiluted SPCS supernatant could be minimized at both dilution levels (Figure 7A). This dilution strategy was also effective on both the Cel6A and Cel7B based ELISA’s and a 100-fold dilution in PBS seemed to consistently give an ELISA signals similar to the PBS control for both Cel6A and Cel7B ELISA (Figure 7B and 7C).

### Can an ELISA be used to follow enzyme distribution during SPCS hydrolysis?

**Figure 8:**
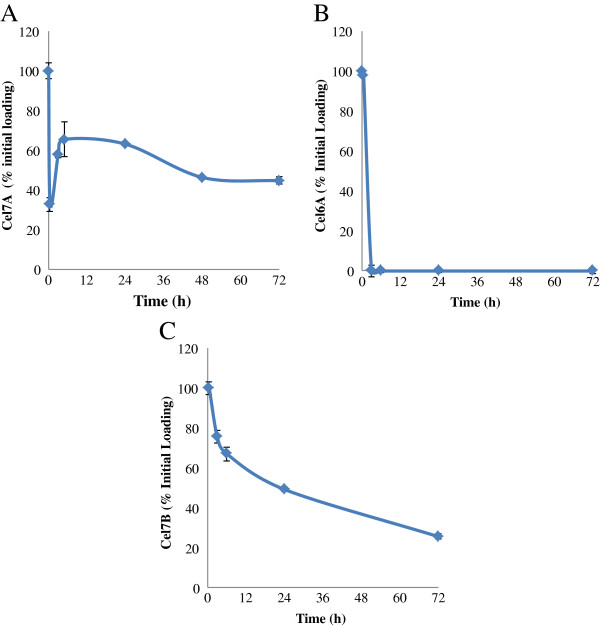
**Adsorption profiles of Cel7A (A), Cel6A (B), and Cel7B (C) during hydrolysis of SPCS as determined by a double-antibody sandwich ELISA.** Samples for Cel7A ELISA were obtained by hydrolyzing SPCS at 2% substrate consistency in 0.05 M Na-acetate buffer pH 5.0 with 20 FPU/ g cellulose Accellerase 1000. Samples for Cel6A and Cel7B ELISA were obtained from SPCS hydrolysis using 20 FPU/ g cellulose Celluclast and 40 CBU/ g cellulose β-glucosidase.

We next wanted to assess if the double-antibody sandwich ELISA could be used to quantitatively monitor the time course of individual enzyme adsorption (Cel7A, Cel6A, and Cel7B) during the hydrolysis of SPCS. It was apparent that all 3 enzymes exhibited different adsorption profiles when incubated with SPCS (Figure 8A, B, and C). Most of Cel7A immediately adsorbed to the SPCS after mixing, leaving only about 30% of Cel7A in the supernatant. After 3 hours of hydrolysis, Cel7A started to desorb back to the supernatant with maximum desorption occurring after 6 hours of hydrolysis with about 65% of the initial Cel7A detected in the supernatant. Over prolonged hydrolysis, the concentration of Cel7A in the supernatant decreased progressively (Figure 8A). This partially reversible adsorption of Cel7A confirmed previous work where a combination of techniques, such as zymogram, SDS-PAGE, and enzyme activity assays, were used to semi-quantitatively determine specific Cel7A adsorption/desorption during SPCS hydrolysis [1].

In contrast, Cel6A directly adsorbed onto the SPCS within the first 3 hours and remained tightly bound throughout the course of hydrolysis (Figure 8B). Previous work that looked at Cel6A adsorption used purified Cel6A due to a lack of a specific assay able to monitor Cel6A in the presence of other enzymes. The irreversible adsorption of Cel6A observed in this study using commercial enzyme mixtures was in a good agreement with this previous work [29].

Compared to Cel7A and Cel6A, the adsorption of Cel7B was more gradual with the amount of Cel7B detected in the supernatant continuously declining over the 72 h hydrolysis (Figure 8C). Prior to developing the ELISA method, we had tried to follow the specific adsorption profile of Cel7B by monitoring its profile as determined by zymograms using CMC and xylan as substrates [1]. The quantitative adsorption profiles obtained using the ELISA profile were in a good agreement with the qualitative results obtained previously using zymograms during the 72 h hydrolysis [1].

## Conclusions

A simple, high-throughput assay that can specifically follow and quantify individual enzymes present in the complex enzyme mixtures that are used to hydrolyse pretreated lignocellulosic substrates was developed and demonstrated. The protocols for an immunoassay using antibodies against Cel7A, Cel6A, and Cel7B were developed with the hope of using the method to follow the distribution of individual enzymes during hydrolysis. A combination of MAb’s and PAb’s, as the respective coating and detecting antibodies, was used to develop a double-antibody sandwich ELISA. This method was able to detect and quantify individual enzymes when present in cellulase mixtures. The assay was sensitive over a range of relatively low enzyme concentration (0 – 1 μg/ml), provided the enzymes were first pH adjusted and/or heat treated to increase their antigenicity. Although lignocellulosic hydrolysates resulted in varying degrees of interference with the assay, the interference could be minimized by diluting the samples in PBS buffer. The immunoassay was employed to quantitatively monitor the adsorption of cellulase monocomponents, Cel7A, Cel6A, and Cel7B that are present in both Celluclast and Accellerase 1000, during the hydrolysis of SPCS. All three enzymes exhibited different individual adsorption profiles. The specific and quantitative adsorption profiles observed with the ELISA method was in agreement with earlier work where more laborious enzyme assay techniques were used.

## Methods and materials

### Purification of cellulase monocomponents, Cel7A, Cel6A, Cel7B, and Cel5A

The cellulase monocomponents Cel7A, Cel6A, Cel7B, and Cel5A were purified from Celluclast (Novozyme) using previously described methods [12,30-32]. The Ninhydrin assay [33] was then used to determine the concentrations of these purified enzymes as well as the commercial enzyme mixtures. Bovine serum albumin (BSA, Sigma) was used as the protein standard.

### Preparation of antibodies and determination of their specificity

MAbs against Cel7A, Cel6A, and Cel7B as well as PAb against Cel7B were a kind gift from Dr. Larry Taylor of the National Renewable Energy Laboratory (NREL). PAbs against Cel7A and Cel6A were prepared commercially by Alpha Diagnostic International, Texas. Briefly, synthetic peptides containing amino acid sequence with high antigenicity from enzymes Cel7A and Cel6A were identified and synthesized The peptide sequence used to raise the Cel7A PAb was R-A-Q-S-A-C-T-L-Q-S-E-T-H-P-P-L-T-W-Q-K, and that for Cel6A PAb was C-D-T-L-D-K-T-P-L-M-E-Q-T-L-A-D-I-R. Following peptide conjugation, antibodies were raised by immunizing rabbits with these peptides. The antibody titers in the rabbit sera and its reactivity to the target peptide were tested using ELISA. Once the test results met the required criteria, the antibody was then purified from the sera by using affinity columns coated with the respective peptide.

The specificity of all MAbs and PAbs were first tested against purified enzymes and enzyme mixtures by using the Western Blot technique following a protocol described by the assay kit producer (Immun-Blot Assay Kit, Bio-Rad). The reactivity and specificity of MAbs against all 3 enzymes (Cel7A, Cel6A, and Cel7B) were tested against purified Cel7A from Celluclast and 3 commercial enzyme mixtures (30 μg each) Accellerase 1000 (Genencor-DuPont), Celluclast, and Cellic CTec 2 (Novozymes). PAbs against Cel7A and Cel6A were tested against purified Cel7A and Cel6A from Celluclast as well as the commercial cellulase mixtures Celluclast and Cellic CTec 2. The specificity and reactivity of PAb against Cel7B were similarly tested against purified Cel7A, Cel6A, and Cel7B from Celluclast as well as the 3 enzyme mixtures Accellerase 1000, Celluclast, and Cellic CTec 2.

Briefly, purified enzymes and enzyme mixtures were separated using sodium dodecyl sulphate polyacrylamide gel electrophoresis (SDS-PAGE) on 4-12% (w/v) Bis-Tris Criterion XT polyacrylamide gels (Bio-Rad). Following electrophoresis, the polyacrylamide gel was equilibrated in the transfer buffer (Towbin buffer containing 25 mM Tris, 192 M glycine, and 20% (v/v) methanol) for 30 minutes. The proteins in the polyacrylamide gel were then transferred to a polyvinylidene difluoride (PVDF) membrane using a Trans-Blot Semi-Dry Electrophoretic Transfer Cell (Bio-Rad) for 60 minutes at 15 V. After washing with Tris-buffered saline containing 0.05% (v/v) Tween 20 (TTBS), the membrane was immersed in Tris-buffered saline (TBS) containing 3% (w/v) gelatin to block any unoccupied sites on the membrane. Antibodies to be tested were then added at a concentration of 5 μg/ml diluted in TTBS containing 1% (w/v) gelatin, and the membrane was incubated for 1 hour. Bound MAbs were detected by immersing the membrane in TTBS-1% (w/v) gelatin containing 1/3000 dilution of goat anti-mouse-IgG antibody conjugated to alkaline phosphatase (GAM-AP, Bio-Rad) for 1 hour whereas bound PAbs were detected by using goat anti-rabbit-IgG antibody conjugated to alkaline phosphatase (GAR-AP, Bio-Rad). After a final wash, the membrane was developed by incubation in the color development/substrate solution containing 5-bromo-4-chloro-3'-indolyphosphate p-toluidine salt (BCIP) and nitro-blue tetrazolium chloride (NBT) for 30 minutes. The reaction was stopped by immersing the membrane in nanopure water for 10 minutes.

### Optimization of double-antibody sandwich ELISA

A double-antibody sandwich ELISA was developed as it was previously shown to have improved specificity for a target cellulase enzyme present in a cellulase enzyme mixture. MAbs were used as the coating antibodies and PAbs as the detecting antibodies to minimize possible interference from other enzymes, sugars and other materials that may be present in the enzyme mixture [23]. Unless otherwise stated, all reagents were added at a volume of 100 μl, and incubation was carried out at 37°C. Maxisorp plates (Nunc) were coated with MAb diluted in 1× phosphate-buffered saline (PBS) pH 7.5 at 4°C overnight. The wells were then washed with PBS and blocked with 2% (w/v) BSA diluted in 1× PBS for 2 hours. After the wells were washed, enzyme standards and/or samples were added to the wells and incubated for 2 hours. As antibody-antigen interaction is optimum at pH > 7 [24], the enzyme samples were added to the wells after dilution in PBS pH 7.5 to ensure that the enzyme samples were in a solution at greater that pH > 7. Purified Cel7A, Cel6A, and Cel7B were serially diluted (concentrations 0–2.5 μg/ml) in PBS to develop standard curves. After incubation with each of the enzymes, the plate was washed, and the PAb, diluted in PBS with 1% (w/v) BSA, was added to each well. The plate was then incubated for 1 hour. Following another washing step, the third antibody, a commercial GAR-AP (Bio-Rad) diluted in PBS with 1% (w/v) BSA, was added to the wells and incubated for another hour. After a final washing step, 35 mg/ml of *p*-nitrophenylphosphate (Bio-Rad), a substrate for alkaline phosphatase (AP), was added to the wells and the plate was incubated at room temperature for 30 minutes or until sufficient colour had developed. Colour development was stopped by adding 400 mM glycine-NaOH. The amount of enzymes bound to the sandwich ELISA was quantified by measuring the absorbance of *p*-nitrophenyl at 405 nm.

### Determining the concentrations of the MAb, PAb, and the enzyme-antibody conjugate

The concentrations of the MAb, PAb, and GAR-AP were optimized for the Cel7A ELISA. Various concentrations of each antibody were tested against a series of concentrations of purified Cel7A. During each antibody optimization, the concentrations of the other two antibodies were kept constant. MAb’s against Cel7A was tested at two different concentrations of 10 and 50 μg/ml. Once the concentration of the MAb was optimized, the PAb against Cel7A was assayed at concentrations of 1.75, 3.5, 7, and 14 μg/ml. Similarly, two different dilutions (1/500 and 1/1750 or 1 and 0.3 μg/ml, respectively) of the third antibody, (the GAR-AP conjugate) were assessed.

### Optimization of sample treatments

As heat treatment had previously been used successfully to improve the sensitivity of an ELISA system for Cel7A [23] we investigate the possible influence of heat treatment on the ELISA when 5 μg/ml of each of the purified enzymes were heated at 100°C for 10 minutes. Each enzyme was heated in either Na-acetate buffer (0.05 M pH 5.0) or in PBS pH 7.5. After cooling the samples to room temperature, the enzymes that had been heated in Na-acetate buffer were first diluted with PBS and then added to the ELISA plate. Samples heated in PBS were directly added to the wells at the same final concentration. Unheated samples were added as controls.

### Determination of the specificity of ELISA

The specificity of each ELISA was determined by comparing the ELISA signal of the target enzyme in the absence and presence of the 3 other cellulase enzymes (Cel7A, Cel6A, Cel7B, and Cel5A). The reconstituted enzyme mixture consisted of 5 μg/ml of the target enzyme and 2.5 μg/ml of each of the other 3 cellulase enzymes in Na-acetate buffer (0.05M, pH 5.0). For Cel7A and Cel6A ELISA, the reconstituted enzyme mixture was heated at 100°C for 10 minutes, serially diluted in PBS to make a standard curve, and then added to the well. Similarly, 5 μg/ml of the pure enzyme sample was subjected to the same treatment. The standard curve obtained from the purified enzyme sample was then compared with that obtained from the reconstituted enzyme mixture. The specificity of Cel7B ELISA was determined in a similar manner except that the enzyme samples were not heated but directly added to the wells after dilution in PBS. The specificity of ELISA was also tested using commercial enzyme mixtures to determine if a dilution of a commercial enzyme mixture can be used to construct a standard curve, obviating the need to use purified enzymes. Commercial enzyme mixtures were diluted in Na-acetate buffer (0.05M, pH 5.0), subjected to the heat treatment when required (i.e. for Cel7A and Cel6A ELISA), serially diluted in PBS, and then added to the wells.

### Lignocellulosic feedstocks and their pretreatment

An agricultural residue (corn stover), softwood (Douglas-fir) and hardwood (hybrid poplar) chips were used as feedstocks and were pretreated by SO_2_-catalyzed steam pretreatment. The pretreatments were performed at near optimal conditions that had previously been determined to provide maximum hemicellulose recovery while ensuring effective enzymatic hydrolysis of the cellulose component (steam pretreatment: corn stover [34], Douglas-fir [35], and poplar [36]). After pretreatment, the cellulose rich water insoluble components were washed, filtered and refrigerated for long-term storage. The details of the pretreatment conditions and the chemical compositions of the pretreated substrates have been described earlier [36,37].

### Influence of lignocellulosic derived components present in the hydrolysis supernatants on the ELISA

Other than the enzymes, lignocellulosic hydrolyzates can contain various materials derived from the biomass such as soluble phenolic compounds that may interfere with the ELISA. Therefore, to try to determine the possible influence of these substrate materials on the ELISA’s, lignocellulosic supernatants obtained from steam pretreated corn stover (SPCS), steam pretreated poplar (SPP), steam pretreated douglas fir (SPDF), and Avicel PH-101 (Sigma), a pure crystalline cellulose substrate, were incubated in 0.05 M Na-acetate buffer pH 5.0 for 24 hours at 50°C with rotational mixing in an incubator (Combi-D24) in the absence of any enzymes. After centrifugation to remove the solid substrate, a known concentration of the target enzyme was added to these supernatants. The same enzyme concentration diluted in 0.05 M Na-acetate buffer pH 5.0 was used as a control. These samples were subjected to heat treatment when required, diluted in PBS and then added to the well. The influence of sugar was not determined as previous work had shown that sugars did not interfere with the ELISA when a MAb was used as the first antibody [23]. As previous work had suggested that “diluting-out” these substrate-derived materials could minimize their interference of the ELISA [20] the supernatants were diluted 10 or 100 times with PBS.

### Enzymatic hydrolysis of SPCS

The enzymatic hydrolysis of SPCS was carried out in 15 ml tubes (Corning) in four replicates at 50°C with a rotational mixing at 20 rpm. The SPCS was diluted to 2% (w/v) solid loading with Na-acetate buffer (0.05 M, pH 5.0) to a total volume of 5 ml. Accellerase 1000 was added at 51 mg protein/g glucan, which corresponded to 20 FPU/g glucan. Similarly, SPCS hydrolysis was also carried out using Celluclast at 20 FPU/g glucan or 52 mg protein/g glucan. Concurrently, SPCS was also incubated in Na-acetate buffer (0.05 M, pH 5.0), in the absence of enzymes, to serve as a substrate alone control (SPCS SC).

During hydrolysis, samples were taken at different time points over a period of 72 hours. After centrifugation, the unbound proteins in the supernatant were recovered by transferring the supernatant into 15 ml tubes. One ml of the supernatant was collected and heated at 100°C for 10 minutes for subsequent glucose measurement using the glucose oxidase assay [38]. The remaining supernatant was stored at 4°C for subsequent ELISA assay using the optimized conditions to determine any changes in Cel7A, Cel6A, and Cel7B concentrations during hydrolysis.

### The development of a double-antibody sandwich ELISA to quantify Cel7A, Cel6A, and Cel7B adsorption during SPCS hydrolysis

ELISA plates were incubated with 10 μg/ml of MAb in PBS at 4°C overnight. The wells were then washed with PBS and blocked with 2% (w/v) BSA diluted in PBS for 2 hours. After the wells were washed, enzyme standards and/or samples were added to the wells and incubated for 2 hours. For the Cel7A and Cel6A ELISA’s, before the addition of samples to the ELISA plate, the purified enzyme samples or the hydrolysate samples were first heated at 100°C for 10 minutes. The heat treatment was always done in Na-acetate buffer (0.05 M pH 4.8). After cooling to room temperature, the samples were diluted in PBS and then added to the ELISA plate. This dilution in PBS not only adjusted the pH of the added samples but also diluted any interfering materials that might be present in lignocellulosic supernatants. After incubation with the enzyme samples for 2 hours, the plate was washed with PBS. A PAb toward the enzyme of interest was added at a concentration of 14 μg/ml diluted in PBS containing 1% (w/v) BSA. The plate was then incubated for 1 hour. Following another washing step, the third antibody, a commercial GAR-AP (Bio-Rad) diluted 1/500 in PBS containing 1% (w/v) BSA, was added and incubated for another hour. After a final washing step, *p*-nitrophenylphosphate (Bio-Rad) was added, and the plate was incubated until sufficient colour had developed. The colour development was stopped by adding 400 mM glycine-NaOH. The amount of enzymes bound to the sandwich ELISA was quantified by measuring the absorbance of *p*-nitrophenyl at 405 nm.

By following this protocol, the amount of Cel7A, Cel6A, and Cel7B present in SPCS hydrolysates (unbound proteins) during 72-hour hydrolysis could be quantified. Purified Cel7A, Cel6A, and Cel7B were used to make standard curves. In each of the ELISA assays, the SPCS SC were included and treated in the same way as the hydrolysate samples, to determine the possible influence of any materials in the hydrolysates. The initial enzyme in buffer without any substrate (enzyme control-EC) was also included, to determine the initial concentration of each enzyme. Protein samples for Cel7A ELISA were obtained from SPCS hydrolysis using 20 FPU/ g cellulose of Accellerase 1000. Those for Cel6A and Cel7B ELISA were obtained from SPCS hydrolyzed by 20 FPU/ g Celluclast complemented with 40 CBU/ g cellulose of β-glucosidase.

## Abbreviations

BSA: Bovine serum albumin; CMC: Carboxymethyl cellulose; ELISA: Enzyme-linked immunosorbent assay; FPLC: Fast Protein Liquid Chromatography; GAM-AP: Goat anti-mouse IgG antibody conjugated to alkaline phosphatase; GAR-AP: Goat anti-rabbit IgG antibody conjugated to alkaline phosphatase; IP: Immune-precipitation; MAb: Monoclonal antibody; PAb: Polyclonal antibody; MW: Molecular weight; PBS: Phosphate-buffered saline; SPCS: Steam pretreated corn stover; SPP: Steam pretreated poplar; SPDF: Steam pretreated Douglas-fir.

## Competing interests

The authors declare that they have no competing interests.

## Authors’ contributions

All authors contributed jointly to all aspects of the work reported in the manuscript. AP carried out much of the laboratory work, contributed to planning, interpretation of results and drafting of the paper. JH contributed to the purified enzymes used in the study. VA contributed to the planning, interpretation and drafting. JS contributed to the planning, interpretation and writing of the manuscript. All authors read and approved the final manuscript.
